# Supplementary material for: The interactome of intact mitochondria by cross-linking mass spectrometry provides evidence for coexisting respiratory supercomplexes
Source: Mol Cell Proteomics. 2017 Dec 8;17(2):216–32. doi: 10.1074/mcp.RA117.000470 (PMC5795388; doi:10.1074/mcp.RA117.000470)
Supplement: Supplemental Data [file supp_RA117.000470_133922_0_supp_23978_fzffwf.zip › spectra_annotation/mito_DR_spectra_annotation/230-1-15-1-4-1.pdf]

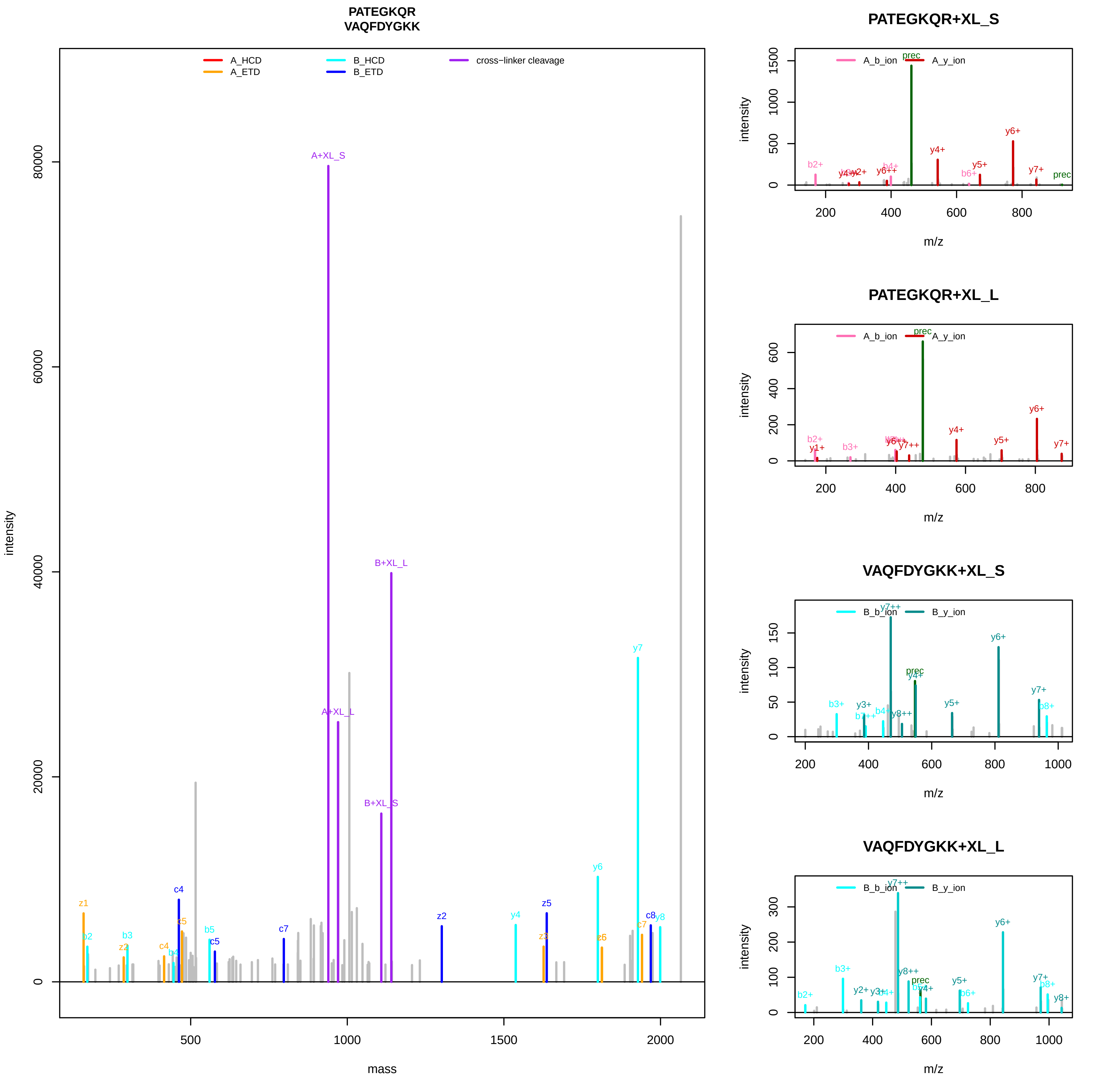
PATEGKQR
VAQFDYGKK
A_HCD
A_ETD
B_HCD
B_ETD
cross−linker cleavage
A+XL_S
B+XL_L
A+XL_L
B+XL_S
y7
y6
y4
z5
z2
c7
c4
c5
b5
b3
b2
z1
z2
c4
b4
z3
z6
c7
c8
y8
intensity
mass
0
20000
40000
60000
80000
500
1000
1500
2000
PATEGKQR+XL_S
A_b_ion
A_y_ion
prec
b2+
y4+
y6+
y5+
b6+
y7+
intensity
m/z
PATEGKQR+XL_L
A_b_ion
A_y_ion
prec
b2+
y1+
b3+
y4+
y5+
y6+
y7+
y7++
VAQFDYGKK+XL_S
B_b_ion
B_y_ion
y7++
y6+
prec
y4+
b3+
y3+
y5+
y7+
b8+
y8++
VAQFDYGKK+XL_L
B_b_ion
B_y_ion
y7++
y6+
b3+
y8++
prec
y5+
b6+
y7+
b8+
y8+
b2+
y2+
y3+
y4+
